# Phage–Antibiotic Synergy Enhances Biofilm Eradication and Survival in a Zebrafish Model of *Pseudomonas aeruginosa* Infection

**DOI:** 10.3390/ijms26115337

**Published:** 2025-06-01

**Authors:** Ling-Chun Lin, Yu-Chuan Tsai, Nien-Tsung Lin

**Affiliations:** 1 Institute of Medical Sciences, Tzu Chi University, No. 701, Sec. 3, Zhongyang Road, Hualien 97004, Taiwan; 111353101@gms.tcu.edu.tw; 2 Master Program in Biomedical Sciences, School of Medicine, Tzu Chi University, No. 701, Sec. 3, Zhongyang Road, Hualien 97004, Taiwan

**Keywords:** phage therapy, *Pseudomonas aeruginosa*, multidrug resistance, biofilm inhibition, phage–antibiotic synergy, zebrafish model

## Abstract

*Pseudomonas aeruginosa* is a gram-negative opportunistic pathogen that poses a significant threat due to its increasing multidrug resistance, particularly in clinical settings. This study aimed to isolate and characterize a novel bacteriophage, phiLCL12, from hospital wastewater and evaluate its potential in combination with antibiotics to combat *P. aeruginosa* infections and biofilm formation. Transmission electron microscopy revealed that phiLCL12 possesses a long contractile tail. The isolated phage exhibited a broad host range of 82.22% and could adsorb up to 98% of its target within 4 min. It was effective against multidrug-resistant strains at both high and low multiplicities of infection (MOIs) levels in lysis tests. Taxonomic classification was determined using PhaGCN2 and Whole genomic analysis, and the results identified phiLCL12 as a member of the *Pbunavirus*. In vitro experiments demonstrated that phiLCL12 significantly enhanced biofilm clearance and inhibited biofilm formation when combined with sub-inhibitory concentrations of imipenem. Furthermore, in vivo experiments using a zebrafish model showed that phage–antibiotic synergy (PAS) improved survival rate compared to antibiotic treatment alone. This study demonstrates that phiLCL12 is effective in both eradicating and preventing *P. aeruginosa* biofilm formation. The combination of phiLCL12 and imipenem provides a synergistic effect, significantly enhancing survival outcomes in a zebrafish model. These findings highlight the potential of phage–antibiotic synergy as a promising therapeutic strategy against biofilm-associated infections.

## 1. Introduction

*Pseudomonas aeruginosa* is a gram-negative opportunistic pathogen commonly found in the environment and is a major cause of nosocomial infections, particularly in hospital settings and wastewater systems [1,2,3,4]. It poses a serious threat to individuals with burns, compromised immune systems, or cystic fibrosis, where infections can lead to severe illness and increased mortality [5,6,7,8]. *P. aeruginosa* is particularly problematic due to its ability to form biofilms, which are structured communities of bacteria embedded in a self-produced extracellular polymeric substance (EPS) [9]. Biofilm formation enhances bacterial resistance to antibiotics and host immune responses, making infections difficult to eradicate. These biofilms are frequently found on medical devices such as catheters, ventilators, and implants, contributing to persistent infections and increased morbidity [10,11,12]. Of particular concern is the rapid rise in multidrug-resistant (MDR) *P. aeruginosa* strains, complicating treatment efforts. Recent data from Taiwan indicate an increase in MDR *P. aeruginosa* isolates, with resistance rates rising from less than 18% in 2015 to 27.5% in 2018 [13,14]. An analysis of 1127 clinical strains collected between 2015 and 2018 revealed that 11% were resistant to carbapenems and 27.5% were classified as MDR, emphasizing the growing challenge in treating these infections [13,15]. Additionally, surveillance data have identified *P. aeruginosa* as a predominant pathogen responsible for severe infections in intensive care units.

The global antibiotic resistance crisis extends beyond *P. aeruginosa*, with the World Health Organization identifying six key bacterial pathogens collectively known as ESKAPE pathogens. These include vancomycin-resistant *Enterococcus faecium*, methicillin-resistant *Staphylococcus aureus*, carbapenem-resistant or extended-spectrum beta-lactamase (ESBL)-producing *Klebsiella pneumoniae*, carbapenem-resistant *Acinetobacter baumannii*, carbapenem-resistant *P. aeruginosa*, and carbapenem-resistant or ESBL-producing *Enterobacter cloacae* complex [16]. The widespread misuse of antibiotics has accelerated the emergence of resistance, diminishing the effectiveness of conventional treatments. The WHO warns that if this trend continues, antimicrobial resistance could lead to as many as 40 million deaths annually by 2050 [17].

Given the urgent need for alternative therapies, bacteriophage (phage) therapy has emerged as a promising strategy to combat MDR bacterial infections. Phages are viruses that specifically target and lyse bacterial cells, offering a potential solution to antibiotic resistance. Several studies have demonstrated the efficacy of phage therapy against MDR bacteria, including *P. aeruginosa*. For instance, phage application has been shown to significantly reduce MDR *P. aeruginosa* contamination on medical equipment and clear biofilms from endotracheal tubes [18,19,20]. However, a major challenge of phage therapy is the potential for bacteria to develop resistance. In recent years, phage–antibiotic synergy (PAS) has gained attention as a more effective approach to overcome this limitation. This strategy enhances bacterial eradication by combining phages with antibiotics, reducing the likelihood of resistance development while improving biofilm clearance [21]. Previous studies have demonstrated that PAS can enhance antibiotic efficacy and outperform traditional treatments in biofilm eradication [22,23].

In this study, we aimed to isolate a novel *P. aeruginosa*-specific phage from environmental samples and assess its potential in combination with antibiotics to inhibit biofilm formation. Additionally, we evaluated the therapeutic efficacy of this phage–antibiotic combination against MDR *P. aeruginosa* infections using an in vivo zebrafish model.

## 2. Results

### 2.1. Isolation, Purification, and Morphology of P. aeruginosa Phage phiLCL12

**Figure 1:**
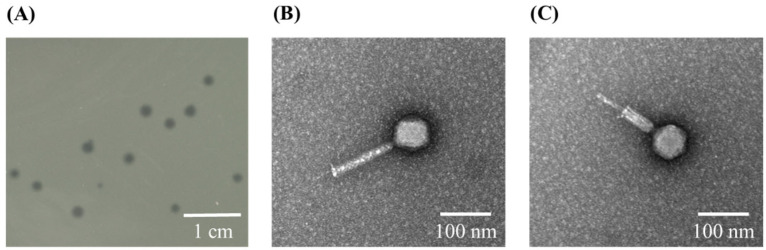
Lytic plaques and morphology of phiLCL12. (**A**) Clear, round lytic plaques observed when using *P. aeruginosa* LCL12 as the host strain; scale bar, 1 cm. (**B**) Transmission electron microscopy (TEM) image of phiLCL12 revealing its morphology, with a head diameter of 71.42 nm and a tail length of approximately 142.85 nm, categorizing it as a long-tailed phage; scale bar, 100 nm. (**C**) Stretching pattern of the phiLCL12 tail observed using TEM; scale bar, 100 nm.

We successfully isolated a phage, phiLCL12, capable of infecting *P. aeruginosa* LCL12, from wastewater near Hualien Tzu Chi Hospital. Spot tests and plaque assays revealed that phiLCL12 produced lytic plaques with an average diameter of 1.2 ± 0.3 mm (*n* = 20), characterized by a clear center and a diffuse, blurry periphery (Figure 1A). To analyze the morphology of phiLCL12, we purified the phage (1 × 10^12^ PFU/mL) using CsCl gradient centrifugation, followed by negative staining with 2% uranyl acetate. TEM showed that the head of phiLCL12 was 71.42 nm in diameter and the contraction tail was about 142.85 nm long (Figure 1B). The stretching pattern of the tail was also observed (Figure 1C). Based on these morphological characteristics, phiLCL12 was a member of long-tailed phages with stretching pattern, which is consistent with a previous study [24].

### 2.2. Host Typing and Sensitivity to phiLCL12

*P. aeruginosa* is a pathogen associated with respiratory, urinary, blood, and burn wound infections [25,26,27] and is a significant clinical challenge due to its ability to infect a variety of tissues. Previous studies have shown that the type III secretion system of *P. aeruginosa* delivers the effector proteins ExoS and ExoU into eukaryotic cells, resulting in severe infections [28]. In this study, we first evaluated the susceptibility of 45 clinical *P. aeruginosa* isolates to phiLCL12 using a dilution spot assay. The results showed that 37 of the 45 strains (82.22%) were susceptible to phiLCL12. We then tested these isolates for the presence of the exoS and exoU virulence genes by PCR and found that only two strains lacked both genes and were resistant to phiLCL12 (Table 1). These findings suggest that phiLCL12 has the potential to be a therapeutic agent for the treatment of virulent *P. aeruginosa* infections.

### 2.3. Biological Characteristics of phiLCL12

**Figure 2:**
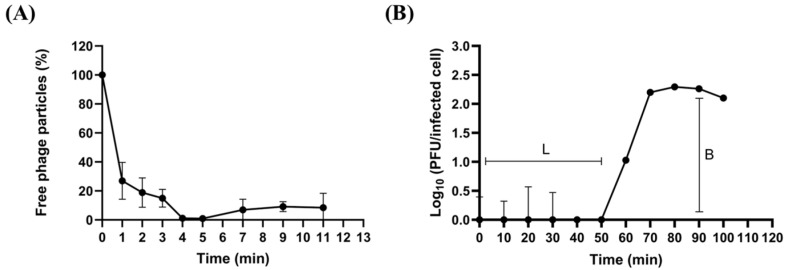

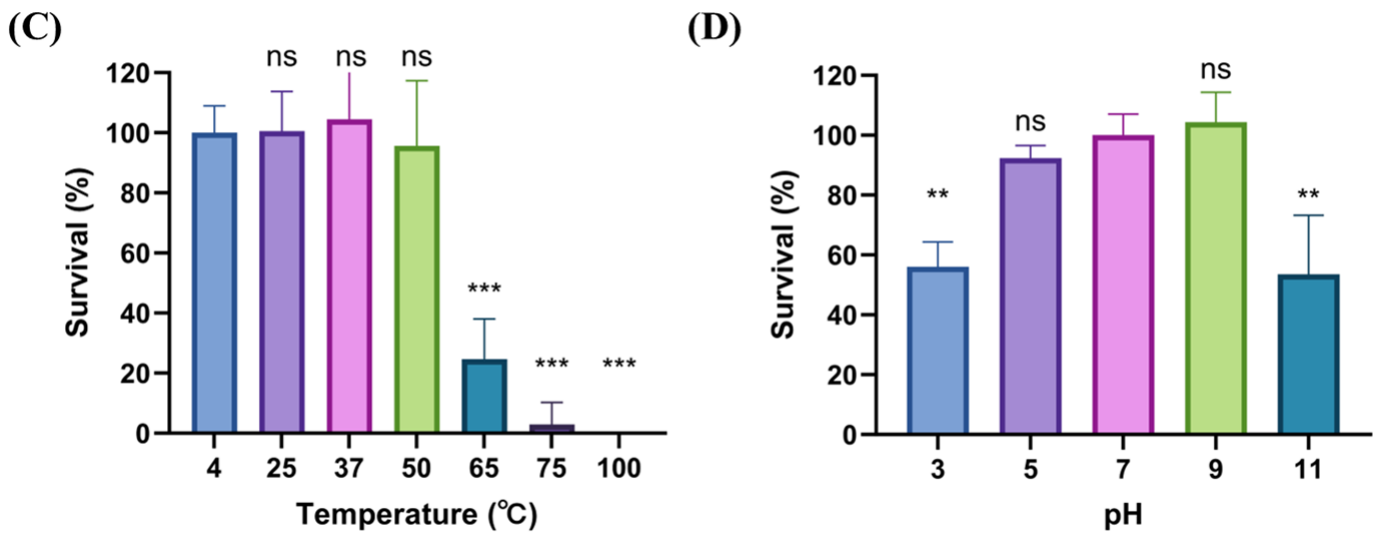
Biological characteristics of phiLCL12. (**A**) Adsorption assay of phiLCL12 conducted at an MOI of 0.0005 using *P. aeruginosa* LCL12 as the host. (**B**) One-step growth curve of phiLCL12 at an MOI of 0.01 in LCL12 culture broth. “L” represents latent time, while “B” denotes burst size. Stability testing of phiLCL12 performed by culturing it at seven different temperatures (**C**) and five pH levels for 1 h (**D**). All results are based on three independent experiments. “***” indicates *p* < 0.001, “**” indicates *p* < 0.01, “ns” indicates not significant.

To evaluate the adsorption efficiency of phiLCL12 on its indicator host, a clinical isolate *P. aeruginosa* LCL12, we measured its adsorption capacity at a multiplicity of infection (MOI) of 0.0005. The results indicated rapid adsorption, with phiLCL12 attaching to 74% of bacterial cells within the first minute and reaching 98% adsorption by the fourth minute (Figure 2A). After adsorption, the phage injects its DNA into the host, initiating replication and assembly, ultimately leading to the release of numerous progenies. A one-step growth curve analysis revealed that phiLCL12 has a latent period of 50 min at 37 °C and a burst size of 182 PFU per infected cell (Figure 2B).

Phage stability is crucial for effective application and long-term storage [29]. To assess the stability of phiLCL12, a fixed concentration of 1 × 10^6^ PFU/10 μL was exposed to seven different temperatures and five pH levels for 1 h, followed by an evaluation of its survival rate. Compared to the control at 4 °C, phiLCL12 exhibited a significant decline in survival at temperatures above 65 °C, with complete inactivation observed at 100 °C (Figure 2C). Under extreme pH conditions, the phage retained 56% viability at pH 3 and 53% viability at pH 11, demonstrating reduced stability in highly acidic or alkaline environments (Figure 2D).

**Figure 3:**
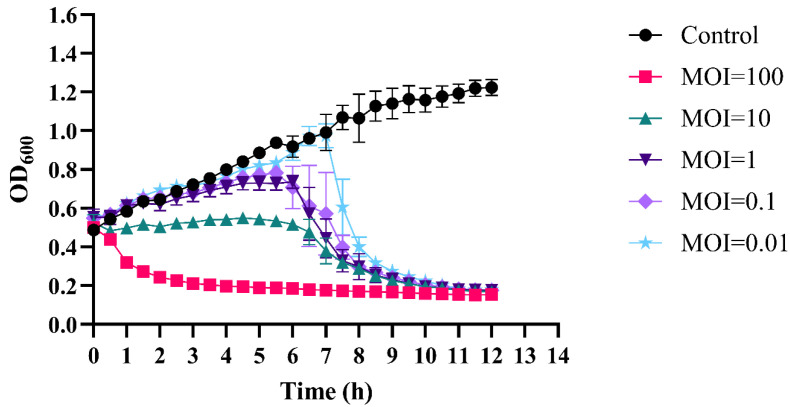
Phage–bacteria growth kinetics at different MOIs. The lytic activity of phiLCL12 against the LCL12 host was evaluated at five different MOIs. Data represent the mean of the measurements recorded every 30 min over a 12 h period, with the standard deviation (SD) indicated using error bars. Each experiment was conducted in triplicate to confirm the reliability of the results.

The effectiveness of phage therapy relies on its ability to lyse the bacterial host at different MOIs [30]. To investigate this, we performed lysis experiments with phiLCL12 in a 96-well plate, inoculating the wells with an initial OD_600_ of 0.5 and applying MOIs of 100, 10, 1, 0.1, and 0.01. The results showed that at an MOI of 100, phiLCL12 efficiently lysed the *P. aeruginosa* LCL12 host, maintaining a consistently low OD value over 12 h. At MOIs of 10, 1, and 0.1, bacterial counts started to decline after six hours, whereas at an MOI of 0.01, a sharp reduction in bacterial density was observed after seven hours (Figure 3). These findings indicate that phiLCL12 produces a substantial number of progenies after infecting the host, allowing even lower MOIs to achieve effective bacterial lysis and population control within 12 h, comparable to higher MOIs.

### 2.4. Genome Annotation and Phylogenetic Analysis

The genome of phiLCL12 was sequenced using NGS, revealing a length of 66,071 bp with a G+C content of 55.7%, which is lower than the typical 66% found in *P. aeruginosa*. Genome annotation via RAST identified 90 ORFs, including 42 hypothetical proteins and 48 proteins with known functions, which were classified into four categories: DNA processing, structural assembly, lysis, and unclassified functions.

**Figure 4:**
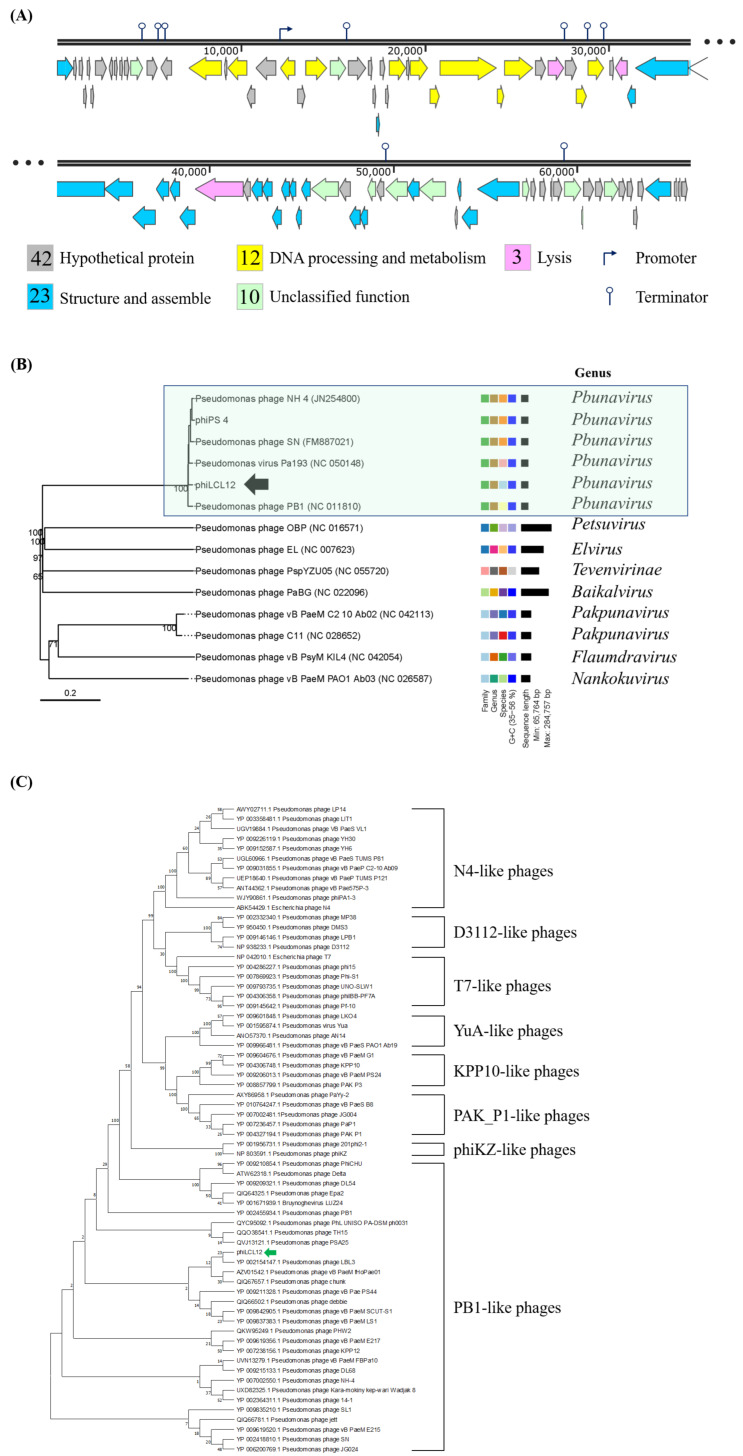
Genome map and phylogenetic analysis of phiLCL12 and related phages. (**A**) Genome map of phiLCL12, with the numbers inside the boxes representing the open reading frames (ORFs). Using BlastP comparisons of protein products, SnapGene 6.0.2 was used to group the phage genome into functional modules, each displayed in a different color. (**B**) Phylogenetic tree of phiLCL12, generated through whole-genome analysis and family classification using VICTOR (https://ggdc.dsmz.de/victor.php# (accessed on 28 March 2022)). Different colors represent various genes and species, with darker blue blocks indicating higher GC content. (**C**) Comparative analysis of terminase proteins from different *Pseudomonas* phages. The genetic relationships were analyzed using MEGA11, with ClustalW employed for sequence alignment, followed by classification with the Neighbor-Joining algorithm. The bootstrap values from 1000 replicates are shown.

Among the DNA-processing proteins, ORF15 (primase), ORF17 (RepB primase), and ORF34 (DNA polymerase III) were identified. Structural proteins included ORF1 (tape-measure protein) and ORF47 (baseplate protein), while lysis-related proteins comprised ORF38 (holin) and ORF51 (lytic transglycosylase). The unclassified proteins featured ORF11 (an IgA Fc receptor) and ORF23 (a TolA protein). ARAGORN analysis confirmed the absence of tRNA and tmRNA genes, while VirulenceFinder 2.0 and ResFinder verified that phiLCL12 does not carry drug resistance or virulence genes [31,32]. Additionally, PhageLead classified phiLCL12 as a strictly lytic phage without temperate genes [33]. Promoter analysis using PhagePromoter identified a promoter in ORF20, while ARNold predicted terminators in ORFs 12, 13, 23, 38, 40, 41, 65, and 77 (Figure 4A) [34,35].

PhaBox prediction by PhaGCN2 program [36] and VICTOR analyses showed that phiLCL12 was classified as *Pbunavirus* (Figure 4B). Phylogenetic analysis of the terminase gene using MEGA11 also clustered phiLCL12 with PB1-like phages, confirming its classification as a member of the PB1-like phage group (Figure 4C).

### 2.5. Structural Protein Analysis

To identify the structural proteins of phiLCL12, we performed SDS-PAGE on CsCl-purified phage particles (1 × 10^11^ PFU/20 μL). Coomassie brilliant blue staining revealed the presence of eight distinct structural proteins ranging in size from 15 to 130 kDa. The most prominent protein band, observed between 40 and 55 kDa, was predicted to correspond to ORF66, which encodes the major head protein. Two other bands were identified by MS/MS spectroscopy as ORF51 (lytic tail protein) and ORF67 (structural protein) (Supplementary File Figure S1).

### 2.6. Phage–Antibiotic Synergy (PAS) Inhibits Bacterial Growth

**Figure 5:**
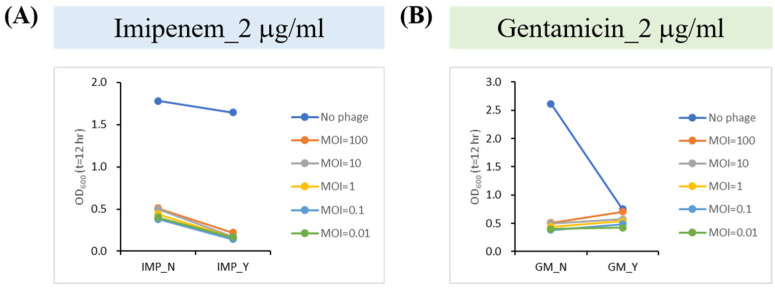
Effect of combining phiLCL12 with different antibiotics. This figure illustrates the effects of phiLCL12 in combination with (**A**) IMP (imipenem), (**B**) GM, gentamicin at different MOIs.; N, no antibiotics added; Y, presence of antibiotics. All results were based on experiments performed in triplicate.

PAS is an emerging therapeutic approach combining phages with antibiotics. This strategy has been shown to produce additive, antagonistic, or synergistic effects depending on the combination used [37]. Previous reports have tested the efficacy of PAS using 1/2 and 1/4 MIC of selected antibiotics combined with phages [38]. Based on the results of the previous drug susceptibility test of LCL12 (Supplementary File Table S1), we selected three antibiotics, imipenem, gentamicin, and ceftazidime, for MIC determination and used them as the basis for determining the antibiotic concentration in the PAS analysis (Table 2). To further investigate the interaction between phiLCL12 and antibiotics, we evaluated the combination of phage with imipenem and gentamicin at different MOIs. The results showed that phiLCL12 exhibited significant synergistic antibacterial activity when combined with imipenem at 1/2 MIC, while it exhibited mixed effects when combined with gentamicin at 1/2 MIC. In addition, the interaction between phiLCL12 and the two antibiotics depended on the MOI (Figure 5).

### 2.7. PAS Outperforms Phage Alone in Eradicating and Inhibiting P. aeruginosa Biofilms

**Figure 6:**
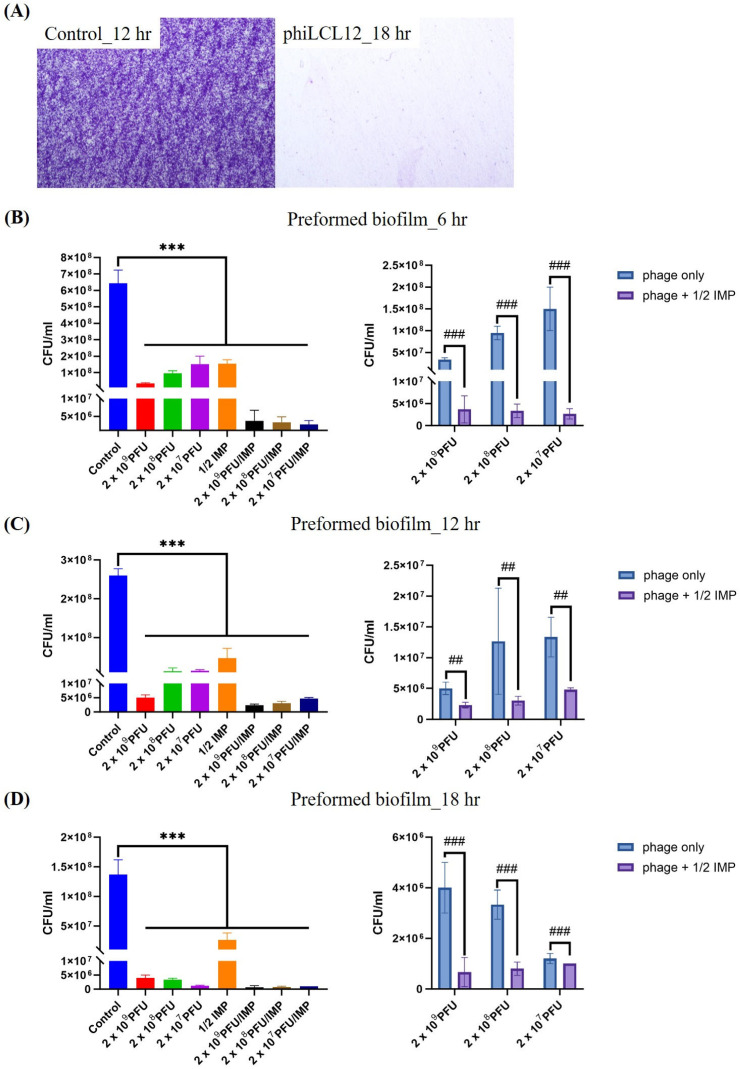
Biofilm clearance by combining phiLCL12 and 1/2 MIC of imipenem over time. After 12 h of *P. aeruginosa* LCL12 biofilm growth at 2 × 10^7^ CFU, varying concentrations of phiLCL12 and 1/2 MIC of imipenem were applied to the experimental groups. (**A**) Qualitative analysis showed no visible biofilms on the coverslips after 18 h of treatment with 2 × 10^9^ PFU of phiLCL12. Quantitative analysis of biofilm clearance over time, with evaluations conducted at 6 (**B**), 12 (**C**), and 18 (**D**) h. All the results were obtained from three independent experiments. On the left panel, *** represents the significance of the control group versus treated groups assessed by one-way ANOVA, *** indicates *p* < 0.001. On the right panel, ## and ### represent the significance of the phage-only group versus the PAS group, which is assessed by two-way ANOVA. “###” indicates *p* < 0.001, “##” indicates *p* < 0.01.

*P. aeruginosa* is a well-established model organism for biofilm research due to its rapid biofilm formation [39]. Previous studies have shown that *P. aeruginosa* LCL12, the host of phiLCL12, reaches peak biofilm production within 12 h (Supplementary File Figure S2). Based on this, biofilms collected at this time point were used for subsequent experiments. We first evaluated the ability of phiLCL12 to remove preformed biofilms using a simulated biofilm model. After 18 h of treatment, no visible biofilm aggregates remained on the coverslips, indicating effective biofilm elimination of phiLCL12 (Figure 6A). With quantitative analysis and one-way ANOVA test at all time points (6 h, 12 h, and 18 h), all treated groups significantly reduced the biofilm cells compared to the untreated control (Figure 6B–D left panel). Furthermore, phage–antibiotic combination treatments generally exhibited more substantial biofilm reduction effects than phage alone treatments, The biofilm eradication and viable count analysis protocols were adapted from those described by Musheer et al. [1], particularly at 6 and 18 h time points (*p* < 0.001). Two-way ANOVA revealed that the addition of 1/2 MIC imipenem significantly reduced biofilm viable counts (*p* < 0.001 at 6 h and 18 h, *p* < 0.01 at 12 h) (Figure 6B–D right panel) over time. Mostly, phage concentration alone showed a significant effect (*p* = 0.0019, 0.2706, 0.0009, respectively, at different time points). This indicates a significant interaction between phage concentration and antibiotic treatment. It means that imipenem at 1/2 MIC enhances biofilm clearance, with the effectiveness positively correlated with phage concentration—the higher the phage concentration, the greater the clearance effect. (Figure 6B–D right panel).

**Figure 7:**
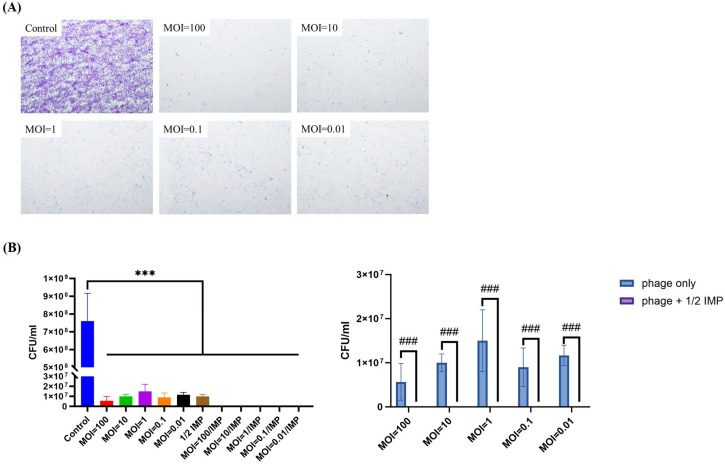
Inhibition of biofilm formation by combining phiLCL12 and 1/2 IMP at various MOIs. *P. aeruginosa* LCL12 at 2 *×* 10^7^ CFU was co-cultured with phiLCL12 at five different multiplicities of infection (MOIs) for 12 h. (**A**) Qualitative analysis shows effective suppression of biofilm formation on coverslips. (**B**) The quantitative analysis supports the qualitative results, demonstrating the extent of biofilm inhibition across different MOIs. All results are from three independent experimental replicates. On the left panel, *** is the control group versus the treated groups assessed by one-way ANOVA, “***” indicates *p* < 0.001. On the right panel, ### is the phage-only group versus the PAS group assessed by two-way ANOVA. “###” indicates *p* < 0.001.

Next, we investigated the ability of phiLCL12 to inhibit biofilm formation. To simulate conditions where phages and bacteria coexist, *P. aeruginosa* LCL12 and phiLCL12 were introduced simultaneously. The results showed no biofilm accumulation on the coverslips at any tested MOI, demonstrating phiLCL12’s strong inhibitory effect on biofilm formation (Figure 7A). Quantitative analysis revealed a similar trend; all co-treated groups showed significant inhibition of biofilm cells compared to untreated control (Figure 7B left panel), with the combination of 1/2 IMP and phiLCL12 achieving superior biofilm inhibition compared to phiLCL12 alone (Figure 7B right panel). Viable cells were scarcely observed following treatment with the combination of 1/2 MIC imipenem and phiLCL12.

### 2.8. In Vivo Assessment of PAS Efficacy in a Zebrafish Model

To assess the toxicity of *P. aeruginosa* LCL12 in zebrafish and determine the optimal bacterial dose for phage therapy, we administered cloacal injections with varying bacterial loads into the zebrafish intestines. The semi-lethal dose (LD_50_) was established by monitoring mortality and symptom progression over 24 h. Zebrafish, 3–3.5 cm in length regardless of sex, were divided into eight groups and observed for three days.

**Figure 8:**
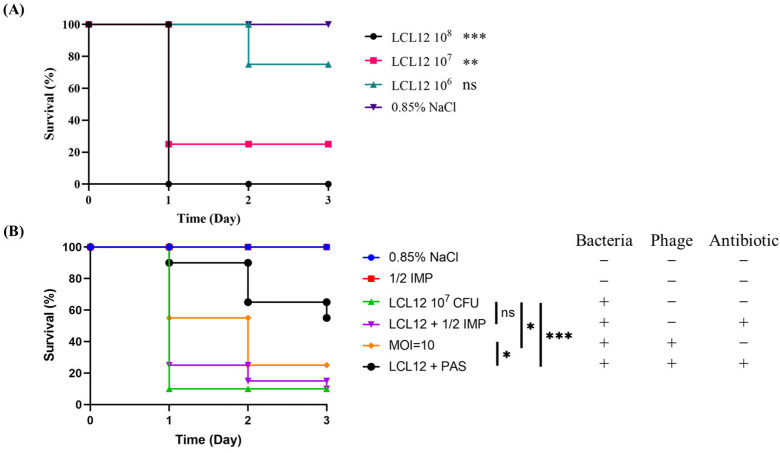
Phage–antibiotic combination therapy outperforms phage therapy alone in zebrafish rescue. (**A**) Evaluation of toxic responses and determination of the semi-lethal dose (LD_50_) in zebrafish over 24 h following cloacal injections of three different *P. aeruginosa* bacterial loads. The results show that an injection of 10^7^ CFU results in the semi-lethal dose being reached within 24 h. (**B**) Survival rates of zebrafish treated with a combination of phiLCL12 and 1/2 MIC imipenem for 3 days assessed using 10^7^ CFU dose as the semi-lethal threshold. “+” indicates the presence of the indicated treatment or component; “–“ indicates its absence. “***” indicates *p* < 0.001, “**” indicates *p* < 0.01, “*” indicates *p* < 0.05. “ns” indicates not significant.

When zebrafish were injected with 1 × 10^8^ CFU/20 µL of *P. aeruginosa* LCL12, five fish developed significant abdominal swelling within 1 h, with mortality beginning at 7 h and reaching 100% within 24 h. The groups injected with 1 × 10^7^ or 1 × 10^6^ CFU/20 µL also showed similar swelling within 1 h. The survival rate in the group injected with 1 × 10^7^ CFU/20 µL dropped to 30% after 24 h. In contrast, the group injected with 1 × 10^6^ CFU/20 µL had a 70% survival rate at 48 h (Figure 8A). These results established that 1 × 10^7^ CFU/20 µL is the LD_50_ within 24 h, making it the optimal dose for subsequent phage therapy experiments. They also confirmed a dose-dependent increase in zebrafish mortality, with higher bacterial loads leading to faster fatalities. Statistical analysis using the log-rank and Gehan–Breslow–Wilcoxon tests revealed significant differences in mortality between the 1 × 10^8^ and 1 × 10^7^ CFU/20 µL groups compared to the control group, which was injected with 0.85% NaCl.

We next evaluated the therapeutic efficacy of phiLCL12 alone and in combination with antibiotics in zebrafish infected with 1 × 10^7^ CFU of *P. aeruginosa* LCL12. Zebrafish were divided into three groups and treated 30 min after bacterial infection with either phiLCL12 at an MOI of 10, 1/2 MIC of imipenem, or a combination of phiLCL12 and 1/2 MIC of imipenem. Survival rates were monitored for three days. The group treated with 1/2 MIC of imipenem had a survival rate of 10%, while the phiLCL12-treated group had a 25% survival rate. However, the combination of phiLCL12 and 1/2 MIC imipenem significantly improved survival, reaching 55% after three days (Figure 8B). These findings suggest that both phage therapy and PAS are effective in reducing *P. aeruginosa* LCL12 infection, with combination treatment showing significantly better outcomes. PAS exhibited a synergistic effect, greatly enhancing the survival of infected zebrafish.

## 3. Discussion

In this study, we successfully isolated and characterized a novel bacteriophage, phiLCL12, which demonstrated stability over a wide range of pH and temperature conditions. Additionally, phiLCL12 exhibited a broad host range, effectively infecting various strains of *P. aeruginosa*, including both invasive and cytotoxic clinical strains. In combination with a sublethal dose of imipenem, phiLCL12 significantly enhanced the eradication and prevention of *P. aeruginosa* biofilm formation compared to phage treatment alone. This combination therapy, known as PAS, also showed remarkable efficacy in rescuing *P. aeruginosa*-infected zebrafish.

*P. aeruginosa* is closely linked to its ability to produce numerous virulence factors such as exotoxins, enzymes, and the type III secretion system (T3SS). T3SS is a key virulence determinant in clinical isolates [40,41]. The *exoS* and *exoU* genes in T3SS are typically mutually exclusive, with *exoS* being more prevalent [40]. However, some studies have identified isolates carrying both genes [42,43]. Among the 45 clinical strains tested in this study, PA18 carried both genes. *P. aeruginosa* strains carrying *exoS* are invasive, while those with *exoU* are cytotoxic. However, the correlation between invasion, cytotoxicity, and clinical disease outcomes remains unclear [44]. Previous studies indicated that *exoU*^+^ strains are more frequently associated with antibiotic resistance than *exoS*^+^ strains and tend to result in more severe clinical manifestations [41,42]. This suggests that most cytotoxic strains are associated with acute infections, highlighting the clinical significance of distinguishing between invasive and cytotoxic strains. Our findings demonstrated the ability of phiLCL12 to infect both invasive (*exoS*^+^) and cytotoxic (*exoU*^+^) strains, broadening its application in clinical settings.

Taxonomy classification by PhaGCN2 program and phylogenetic analysis identified phiLCL12 as a member of the PB1-like phages, which belong to the *Pbunavirus* and represent the largest group of *P. aeruginosa* phages. PB1-like phages are known for their broad host range and ability to infect *P. aeruginosa* strains isolated from the lungs of patients with cystic fibrosis [45,46]. These phages utilize lipopolysaccharides as host receptors [46]. Therefore, it is plausible that phiLCL12 employs a similar attachment mechanism. However, further studies are required to confirm this hypothesis.

Compared to similar PB1-like *Pseudomonas* phage DRL-P1 [47], which featured fair rapid adsorption (~5 min), shorter latency (~30 min), but smaller burst size (~100 PFU vs. ~184 PFU per infected cell), the latent period of phiLCL12 is extended more (50 min) but not uncommon. Several reports showed extended latent (incubation) period of *Pseudomonas* phages [48,49,50]. In a study of ZCPA1 phage [48], its incubation period was about 90 min, which is relatively long. Studies have shown an inverse relationship between bacterial concentration and phage incubation period; that is, at low bacterial concentrations, the incubation period is more extended. In addition, the study also explored the effects of different MOIs (multiplicities of infection) on bacterial growth and found that higher MOIs (such as 10 and 100) could significantly inhibit bacterial growth. Another study investigated the performance of bacteriophages ACQ and UT1 [51] under different nutritional states. In hosts in the exponential growth phase, the average incubation period was 65 min for ACQ and 90 min for UT1. In contrast, in starvation conditions, the incubation period was prolonged to 210 min for ACQ and 165 min for UT1. This suggests the phage incubation period is significantly prolonged when the host is undernourished. The incubation period of the vB_Pae_PLY phage was approximately 40 min [49], which was not considered an extremely long incubation period. However, its burst yield was as high as 853 PFU/infected cell, indicating a strong lytic capacity. The phage was able to lyse 60% of clinical isolates and has potential therapeutic applications. Notably, the latent period of phiLCL12 was 50 min, but lysis ability is strong in both high and low MOIs. Moreover, the inhibition could sustain for hours (Figure 2 and Figure 3). This indicates the comparable effect of phiLCL12 to ZCPA1 and other *Pseudomonas* phages, although with extended latent period. Apparently, a longer incubation period does not affect the burst size or lysis ability of individual phages. It is more likely the result of the interaction between the phage and the host’s physiological conditions. Phages with long latent periods produce more progeny per infection, offering advantages in biofilm control and chronic infections due to sustained action and environmental stability. Previous reports about four *P. aeruginosa* phages—ZCPA1, ACQ/UT1, vB_Pae_PLY, and ASP23 [48,49,50,51]—exhibit distinct biological characteristics suited for different applications. With their long latent periods, ZCPA1 and ACQ/UT1 are better suited for environments where slow, sustained action is advantageous, such as chronic or nutrient-limited infections. In contrast, vB_Pae_PLY, with its high burst size and moderate latent period, shows strong potential for clinical treatment of acute infections. ASP23, with a short latent period and demonstrated efficacy in animal models [50], is ideal for rapid-response therapeutic strategies. Each phage offers unique strengths for tailored phage therapy applications.

*P. aeruginosa* is notorious for its adaptability and ability to form biofilms, contributing to its persistence in both environmental and clinical settings. Studies on using *Pseudomonas* phages for biofilm removal, both as standalone treatments and in combination with other agents (such as phage–antibiotic synergy), revealed different outcomes and application potential [48,52,53,54,55]. In phage monotherapy, phage ZCPA1 demonstrated significant biofilm reduction (~95%) at higher multiplicities of infection (MOIs ≥ 0.1). The efficacy is attributed to the phage’s production of depolymerases and lysins, which degrade the biofilm matrix and bacterial cell walls, respectively [48,52]. Phage AZ1 achieved approximately 99.9% reduction in 48 h old biofilms [52]. The phage’s natural ability to penetrate and disrupt biofilms was highlighted, though complete eradication may require combination therapies. In a phage cocktail (Pa193, Pa204, Pa222, Pa223) study, targeting *P. aeruginosa* isolates from chronic rhinosinusitis patients, individual phages reduced biofilms by 53–73%, while the cocktail achieved an 89% reduction. The enhanced efficacy is due to the expanded host range and prevention of phage-resistant mutants [55]. In our case, phiLCL12 showed biofilm reduction at MOI ≥ 0.01 (Figure 6D), which is better than ZCPA1. PhiLCL12 could remove nearly all preformed biofilm at 18 h standalone treatment (Figure 6A); when combined with 1/2 imipenem, the efficacy of removal (Figure 6B,C) and reduction (Figure 6D) of biofilm showed significant enhancement with comparable efficacy to phage AZ1 but shortened the eradiation time, indicating that phiLCL12 cable as a potential agent for application.

How PAS work together to enhance their individual activity has been studied extensively. In a comprehensive review of PAS, Abedon (2020) [22] listed the mechanisms of PAS, including (1) antibiotic-induced bacterial filamentation enhancing phage replication, (2) phage-mediated degradation of biofilm matrices, improving antibiotic penetration, (3) reduction in the emergence of phage or antibiotic-resistant bacterial mutants, and (4) alterations in bacterial physiology increasing susceptibility to both agents. In a pilot study, Comeau and colleagues introduced the term “Phage–Antibiotic Synergy” (PAS) upon observing that sublethal concentrations of certain antibiotics, notably β-lactams and quinolones, significantly enhanced the production of virulent phages in *Escherichia coli*. This effect was attributed to antibiotic-induced bacterial filamentation, which promoted increased phage replication and accelerated lysis. Importantly, the PAS phenomenon was found to be independent of the bacterial SOS response and consistent across various phage–host systems, suggesting that this synergistic effect has broad applicability [56]. Uchiyama et al. evaluated the synergistic effects of phage–antibiotic combinations against *P. aeruginosa*. Phage KPP22, belonging to the *Pbunavirus*, exhibited the most extensive PAS when combined with anti-pseudomonal antibiotics such as piperacillin and ceftazidime. The findings underscore the importance of selecting appropriate phage–antibiotic pairs to maximize therapeutic efficacy against *P. aeruginosa* infections [57]. Kim et al. investigated the interaction between antibiotics and phage-induced lysis and found that sublethal concentrations of antibiotics could delay bacterial lysis and thus increase phage replication within filamentous bacterial cells. This delay enhances the size of the phage burst, helping to eliminate the bacteria more effectively. The research highlights the role of antibiotic-induced stress responses in modulating phage life cycles [58]. For phiLCL12, we found the plaque size increased around the discs of imipenem, indicating the increased burst size and the interference of the phage production cycle. Imipenem is a beta-lactam antibiotic belonging to the subgroup of carbapenems. We believe phiLCL12 is more similar to the results combined with those of Comeau et al. [56] and Kim et al. [58]. However, we did not check the antibiotic-induced bacterial filamentation. Moreover, the biofilm reduction showed a significant reduction when comparing the combined imipenem and phage-alone group (Figure 6D). This also demonstrated the significant enhancement of the phage-mediated degradation of biofilm matrices under test conditions. Further experiments will be designed to verify the possible PAS mechanism of phiLCL12.

Despite the promising findings of our study, several limitations should be addressed. First, although our biofilm eradication assays demonstrated the efficacy of phiLCL12, they did not fully replicate the complexity of biofilms found in clinical settings. Clinical biofilms such as those on tracheal devices are more intricate and resilient. Future research should focus on simulating these clinical conditions to better understand the potential of phage therapies in real-world scenarios. Second, although zebrafish models are valuable tools for studying infectious diseases, they do not entirely replicate mammalian immune responses. Future studies using mammalian models are necessary to further explore the therapeutic potential of PAS and assess its efficacy against MDR *P. aeruginosa* in clinically relevant settings.

## 4. Materials and Methods

### 4.1. Bacteria Collection and Culture

Bacterial cultures were grown on Lysogeny Broth (LB; BIO BASIC, Markham, CA, USA) Miller agar plates, and incubated overnight in a shaking incubator at 37 °C. Bacterial stocks were stored at −80 °C in 15% glycerol. The phage-indicating host *P. aeruginosa* LCL12 was isolated from a patient’s oral cavity and identified using 16S ribosomal RNA and *P. aeruginosa*-specific primers. Additional clinical strains were used for host-range testing. To determine whether clinical isolates of *P. aeruginosa* exhibited invasive or cytotoxic phenotypes, colony PCR was performed on 45 isolates using the primers described by Horna et al. [42,59]. The sequences for *exoS* and *exoU* were *exoS*-F: 5′GCGAGGTCAGCAGAGTATCG3′, *exoS*-R: 5′TTCGGCGTCACTGTGGAT3′; *exoU*-F: 5′CCGTTGTGGTGCCGTTGAAG3′, and *exoU*-R: 5′CCAGATGTTCACCGACTCGC3′. A fresh colony was resuspended in 100 μL of sterile water and 5 μL was added to a 25 μL PCR mixture containing buffer, primers, dNTPs, and *Taq* polymerase. PCR conditions included initial denaturation (94 °C, 2 min), 36 cycles (94 °C, 30 s; 58 °C, 30 s; 68 °C, 1 min), and a final extension (68 °C, 7 min), followed by cooling (4 °C). The amplicons were analyzed by 2% agarose gel electrophoresis, and the expected sizes were 118 bp (*exoS*) and 134 bp (*exoU*).

### 4.2. Isolation and Purification of Bacteriophage

Wastewater samples from the hospital were filtered (0.45 µm PVDF membrane) and stored at 4 °C. The filtered samples were mixed with mid-log phase *P. aeruginosa* LCL12 (1 × 10^8^ CFU/mL) and incubated at 37 °C for 24 h. Phages were separated by centrifugation (12,000 rpm, 10 min, 4 °C), and the supernatants were spotted onto double-layer LB agar for plaque assays. The plaque assay was repeated three times to confirm the purity of the phage. Phages were enriched and purified using cesium chloride (CsCl) density gradients (1.7, 1.5, 1.45, 1.15 g/mL) and ultracentrifugation (154,300× *g*, 3 h, 4 °C). Purified phages were extracted and dialyzed against ddH_2_O.

### 4.3. Transmission Electron Microscopy (TEM) Analysis

For TEM analysis, the purified phages were sent to the Electron Microscopy Center at Tzu Chi University. A 10 µL phage sample (1 × 10^10^ PFU/mL) was applied to a copper grid and stained with 2% uranyl acetate, then examined using a HITACHI H-7500 transmission electron microscope (Tokyo, Japan).

### 4.4. Host Range Test

The phage concentration was adjusted to 1 × 10^8^ PFU/mL and serially diluted tenfold using SM buffer. Spot tests were performed according to the standard procedures for a variety of clinical *P. aeruginosa* isolates [60]. The plates were incubated at 37 °C for 16–18 h, and the results were recorded.

### 4.5. Adsorption, One-Step Growth Curve, and Stability Assay

For adsorption assays, overnight cultures of *P. aeruginosa* were refreshed in 50 mL of LB and grown to mid-log phase (1.25–1.6 × 10^8^ CFU/mL) at 37 °C. Phages were added at a multiplicity of infection (MOI) of 0.0005. Samples (1 mL) were collected every 1–2 min for 11 min, centrifuged at 12,000 rpm for 1 min each time, and stored on ice. For the one-step growth curve, bacteria were refreshed in 1 mL of LB, grown to the mid-log phase, and then infected with phages at an MOI of 0.01. After adsorption on ice for 15 min, samples were centrifuged and resuspended in fresh LB medium. Samples were collected every 10 min for plaque assay analysis. For stability assays, phages (1 × 10^6^ PFU) were incubated in 1 mL of LB with varying pH (3, 5, 7, 9, and 11) or at different temperatures (4–100 °C) for 1 h. Spot and plaque assays were performed to assess phage titer.

### 4.6. Bacterial Lysis

Overnight cultures of *P. aeruginosa* were refreshed in 50 mL LB and incubated at 37 °C for 3–4 h until the OD_600_ reached 0.5. The bacterial culture was divided into 200 µL aliquots in a 96-well plate, ensuring consistent OD_600_ values. Phages were added at various MOIs (100, 10, 1, 0.1, and 0.01) for infection, with the SM buffer serving as a control. OD_600_ was measured every 30 min for 12 h using a microplate reader (BMG CLARIOstarPlus, Ortenberg, Germany).

### 4.7. Phage DNA Extraction

Phage DNA was extracted using the phenol/chloroform method. Phages (5 × 10^11^ PFU in 500 µL) were mixed with SDS, EDTA, and Proteinase K, then incubated at 55 °C for 3 h with shaking, followed by 65 °C for 15 min. After phenol/chloroform (1:1) and chloroform-only extractions, DNA was precipitated with sodium acetate and ethanol at −20 °C for 16–18 h. The pellet was centrifuged, washed with 75% ethanol, and resuspended in TE buffer. DNA quality and quantity were assessed via 0.7% agarose gel electrophoresis and a NanoDrop spectrophotometer. DNA was stored at −20 °C.

### 4.8. Phage DNA Sequencing and Genome Analysis

Phage phiLCL12 was sequenced by the AllBio company in Taiwan. Genome sequence was annotated by RAST [61]. For genome analysis, gene and protein similarities were searched using NCBI BLAST (https://blast.ncbi.nlm.nih.gov/Blast.cgi) (accessed on 28 March 2022), and gene mapping was performed using the SnapGene Viewer (https://www.snapgene.com/snapgene-viewer (accessed on 30 March 2022)). The conserved regions of each open reading frame (ORF) were analyzed using the NCBI Conserved Domains database (https://www.ncbi.nlm.nih.gov/Structure/cdd/wrpsb.cgi (accessed on 15 April 2022)).

Transfer RNAs (tRNAs) were detected using tRNAscan-SE [62], whereas ribosomal RNAs (rRNAs) were detected using RNAmmer [63]. ARAGORN was used to detect tRNA and tmRNA in the phage genome, while drug resistance and virulence factor genes were predicted using VirulenceFinder 2.0 and ResFinder 3.2. PhageLeads predicted phage life history and PhagePromoter was used for promoter prediction [35]. Terminator predictions were performed using Rho-independent transcription terminators in ARNold [34]. The Terminase large subunit was compared with sequences from various phages using MEGA11 software, employing the Neighbor-Joining method with 1000 bootstrap replications. Genome-based phylogeny and classification of prokaryotic viruses were performed using VICTOR [64]. Taxonomy classification was conducted by PhaBox using PhaGCN2 program [36].

### 4.9. Phage Structure Protein Analysis

Phage samples (1 × 10^10^–1 × 10^11^ PFU) were mixed with 3× sample buffer, boiled for 15 min, shaken for 1 min, and cooled on ice for 10 min. The samples were separated on a 4–20% gradient SDS gel at 120 V for 90 min and stained with Coomassie Brilliant Blue. Protein bands visible on the gel were excised and subsequently identified by tandem mass spectrometry (MS/MS) (Advanced Instrumentation Center of the Department of Medical Research, Hualien, Taiwan).

### 4.10. Phage–Antibiotic Synergy Assay

Overnight bacterial cultures were refreshed in 50 mL of LB and incubated at 37 °C for 3–4 h until the OD_600_ reached 0.5. A 200 µL aliquot of the bacterial suspension was added to each well of a 96-well plate to ensure consistent OD_600_ values across the wells. Phages were added at different infection ratios (MOIs of 100, 10, 1, 0.1, and 0.01), along with antibiotics (1/2 MIC of imipenem or 1/2 MIC of gentamicin). The control group was administered an equal volume of SM buffer. OD600 was measured every 30 min using a full-wavelength microplate reader (BMG CLARIOstarPlus), with the plate returned to the 37 °C incubator between measurements. This process was repeated for 12 h to assess the lysis capacity of the phage–antibiotic combinations.

### 4.11. Biofilm Eradication and Inhibition

The biofilm eradication and viable count analysis protocols were adapted from those described by Musheer et al. [65]. For biofilm eradication, a 22 × 22 mm square coverslip was placed in a 6-well plate containing 2 mL of LB medium and inoculated with *P. aeruginosa* LCL12 (2 × 10^7^ CFU). The plates were incubated at 37 °C for 12 h, with three identical plates used for each condition to ensure reliability and reproducibility. After this initial incubation, phiLCL12 (2 × 10^9^ PFU) or 1/2 the minimum inhibitory concentration (MIC) of imipenem was added to the experimental groups, while the control group received no treatment. The plates were then incubated for an additional 18 h. Following incubation, the supernatant and floating biofilms were removed, and the wells were washed with PBS. The biofilms were stained with 1 mL of 0.1% crystal violet for 30 min, rinsed with ddH_2_O, and observed under a 100× optical microscope (Nikon ECLIPSE 80i, Melville, NY, USA). For viable counts, the coverslip was collected at 6h, 12 h, and 18h, respectively, and the cultures were resuspended with 1 ml PBS and measured by OD600. Then, bacteria were serially diluted 10-fold and plated.

For biofilm inhibition, coverslips were placed in 6-well plates with 2 mL LB medium and inoculated with 2 × 10^7^ CFU of *P. aeruginosa* LCL12. PhiLCL12 at various MOIs was added to the experimental groups, whereas the control group received equal volumes of SM buffer. After 12 h of incubation at 37 °C, the wells were washed, stained with 0.1% crystal violet, and observed under a light microscope. For viable counts, the coverslip was resuspended with 1 ml PBS, and the cultural density was by OD600. Then, bacteria were serially diluted 10-fold and plated.

### 4.12. In Vivo Assessment of PAS Efficacy Against P. aeruginosa LCL12 Infection

Wild-type AB Zebrafish (*Danio rerio*) lines (mixed female and male populations) were housed at the Tzu Chi University Fish Core Facility following standard protocols. Fish were maintained in 9 L tanks at 28 °C under a 14 h light/10 h dark cycle. The infection model was modified from previous studies [66]. *P. aeruginosa* LCL12 was cultured to mid-log phase (2 × 10^8^ CFU/mL), centrifuged at 12,000 rpm for 30 min, and the pellet was resuspended in 1 mL of 0.85% NaCl to achieve a final concentration of 2 × 10^10^ CFU/mL. Adult zebrafish (approximately four months old; *n* = 20 fish per group) were anesthetized with 0.2% tricaine and injected with 20 µL of *P. aeruginosa* LCL12 (2–2.6 × 10^7^ CFU) via the cloaca. Thirty minutes post-infection, the zebrafish were re-anesthetized and injected with either 20 µL phiLCL12 (2–2.6 × 10^8^ PFU) or 1/2 MIC imipenem. The zebrafish were then returned to their tanks and survival was monitored every 2 h for three days. Survival rates were calculated to assess treatment efficacy. All procedures were performed by trained scientists and were approved by the Institutional Animal Care and Use Committee of Tzu Chi University, Hualien, Taiwan (approval no. 111091-A).

### 4.13. Statistical Analysis

Except for survival rate and biofilm quantification analysis, all experimental results were analyzed using *t*-tests. Zebrafish survival rates were analyzed using log-rank and Gehan–Breslow–Wilcoxon tests using GraphPad Prism 10.1.2. Biofilm quantification analysis was conducted using one-way ANOVA and two-way ANOVA by GraphPad Prism 10.1.2. A *p*-value of <0.05 was considered significant.

### 4.14. Nucleotide Sequence Accession Number

The annotated genome sequence has been submitted to GenBank under the accession number OQ428192.

## 5. Conclusions

In summary, we successfully isolated *P. aeruginosa* phage phiLCL12 from wastewater near a hospital and characterized it through comprehensive biological analyses. Our findings indicate that phiLCL12 exhibits a broad host range, effectively removes existing biofilms, and prevents biofilm formation. Moreover, when combined with imipenem, phiLCL12 demonstrated a synergistic effect and successfully rescued zebrafish from *P. aeruginosa* infections. These results highlight the potential of phiLCL12 as a promising therapeutic option for combating multidrug-resistant *P. aeruginosa*.

## Figures and Tables

**Table 1:** Host range and strain typing of phiLCL12.

	Sources	phiLCL12 ^a^	exoS ^b^	exoU ^c^	Phenotype
*P. aeruginosa*					
PA001	clinical	+++	+	−	Invasion
LCL12	clinical	++++	−	+	Cytotoxic
LCL13	clinical	++++	−	+	Cytotoxic
LCL14	clinical	++++	−	+	Cytotoxic
PS-1	bile	+++	+	−	Invasion
PS-2	pus	+++	−	+	Cytotoxic
PS-3	urine	−	+	−	Invasion
PS-4	pus	++++	−	+	Cytotoxic
PS-5	sputum	++	−	+	Cytotoxic
PS-6	sputum	+++	−	+	Cytotoxic
PS-7	sputum	+	+	−	Invasion
PA10	clinical	++	+	−	Invasion
PA13	clinical	−	+	−	Invasion
PA18	clinical	++++	+	+	Invasion/Cytotoxic
PA20	clinical	++++	+	−	Invasion
PA22	clinical	+	+	−	Invasion
PA25	clinical	++	+	−	Invasion
PA75	clinical	++	+	−	Invasion
PA76	clinical	+++	+	−	Invasion
PA77	clinical	++	−	+	Cytotoxic
PA78	clinical	++	+	−	Invasion
PA79	clinical	+++	−	+	Cytotoxic
PA80	clinical	++	+	−	Invasion
PA81	clinical	+	+	−	Invasion
PA82	clinical	++	−	+	Cytotoxic
PA83	clinical	++++	−	+	Cytotoxic
PA84	clinical	+	−	+	Cytotoxic
PA85	clinical	−	−	+	Cytotoxic
PA86	clinical	−	−	−	-
PA87	clinical	+++	+	−	Invasion
PA88	clinical	++	−	+	Cytotoxic
PA89	clinical	+++	+	−	Invasion
PA90	clinical	+++	+	−	Invasion
PA91	clinical	+++	−	+	Cytotoxic
PA92	clinical	+++	+	−	Invasion
PA005	oral	−	+	−	Invasion
PA006	oral	−	−	−	-
PA009	oral	+	+	−	Invasion
PA010	oral	−	+	−	Invasion
PA011	oral	−	+	−	Invasion
PA022	oral	+	+	−	Invasion
PA023	oral	+	+	−	Invasion
PA024	oral	+	+	−	Invasion
PA025	oral	+++	+	−	Invasion

^a^ The symbol ‘+’ indicates the presence of a clear zone at a 10^2^-fold dilution, ‘++’ at a 10^4^-fold dilution, ‘+++’ at a 10^5^-fold dilution, and ‘++++’ at a 10^6^-fold dilution, whereas ‘−’ denotes the absence of any observable clear zone. ^b,c^ The symbol “+” indicates the presence of an amplicon after the PCR reaction, while “−” indicates the absence of an amplicon.

**Table 2:** MIC of *P. aeruginosa* LCL12 for different antibiotics.

	Antibiotic (Sensitivity ^a^)		
	Imipenem	Gentamicin	Ceftazidime
LCL12	4 μg/mL (S)	4 μg/mL (S)	256 μg/mL (R)

^a^: Drug sensitivity: S indicates sensitive; R is resistant.

## Data Availability

Data will be made available on request.

## Supplementary Materials

The following supporting information can be downloaded at: https://www.mdpi.com/article/10.3390/ijms26115337/s1.
