# Supplementary material for: Phage–Antibiotic Synergy Enhances Biofilm Eradication and Survival in a Zebrafish Model of Pseudomonas aeruginosa Infection
Source: Int J Mol Sci. 2025 Jun 1;26(11):5337. doi: 10.3390/ijms26115337 (PMC12155105; doi:10.3390/ijms26115337)
Supplement: Supplementary file 1 [file ijms-26-05337-s001.zip › ijms-3601785-supplementary.pdf]

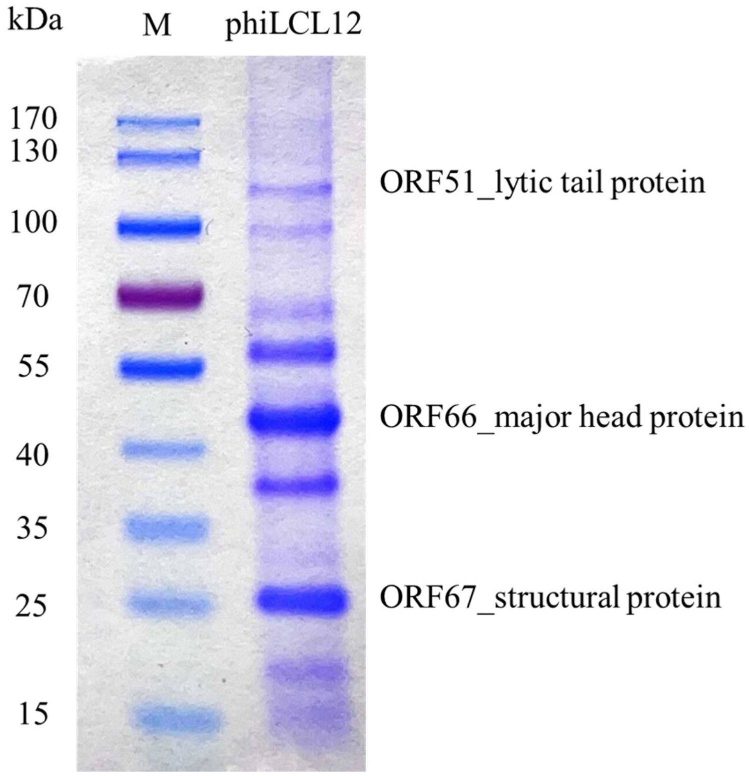

Fig. S1. The structural protein of phiLCL12. A 4–20% gradient gel was used to analyze the structural proteins of phiLCL12. The identified proteins in the image were confirmed using tandem mass spectrometry. M represents the prestained protein marker plus from Protech.

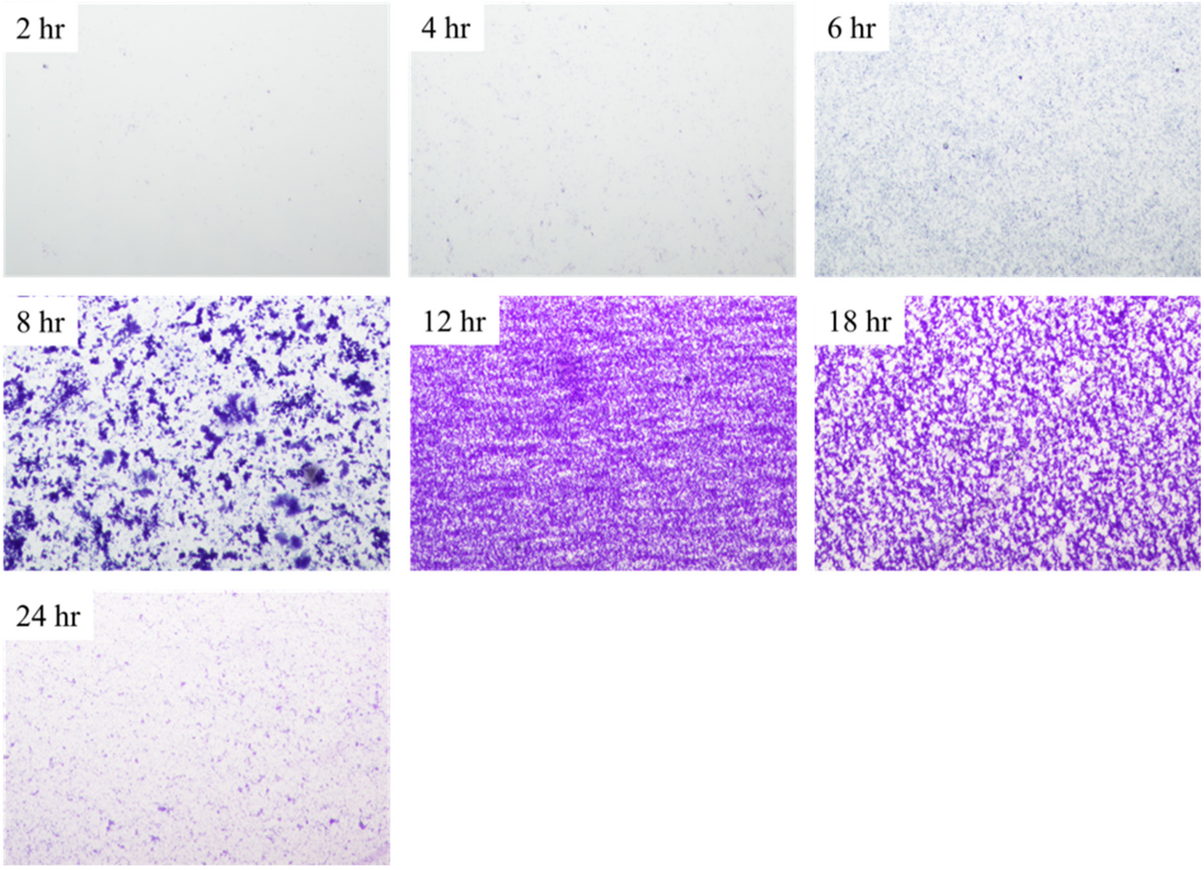

Fig. S2. The dynamic changes in biofilm formation by *P. aeruginosa* LCL12 within 24 hours peaked at 12 hours, followed by a gradual decline.

Table S1. Antibiotics susceptibility profile of *P. aeruginosa* LCL12

Antibiotic	LCL12
Imipenem	S
Meropenem	S
Doripenem	S
Streptomycin	R
Gentamicin	S
Kanamycin	R
Ciprofloxacin	S
Levofloxacin	S
Colistin	S
Ceftazidime	R
Aztreonam	S
Piperacillin/tazobactam	S
Chloramphenicol	R
Tetracycline	R

S: Sensitive, R: Resistant
